# Obstetric Observatory BRAZIL - COVID-19: 1031 maternal deaths because of COVID-19 and the unequal access to health care services

**DOI:** 10.6061/clinics/2021/e3120

**Published:** 2021-06-23

**Authors:** Rossana Pulcineli Vieira Francisco, Lucas Lacerda, Agatha S. Rodrigues

**Affiliations:** I Disciplina de Obstetricia, Departamento de Obstetricia e Ginecologia, Faculdade de Medicina FMUSP, Universidade de Sao Paulo, Sao Paulo, SP, BR; II Departamento de Estatistica, Universidade Federal do Espirito Santo, Vitoria, ES, BR

In addition to the diversity of clinical manifestations and difficulties in managing the disease, coronavirus disease (COVID-19) brought to light inequalities between countries, especially with regard to the accessibility to healthcare services. The worldwide differences in the rates of COVID-19-induced mortality in pregnant and postpartum women reflect the differences in maternal death rates between countries observed before the pandemic. Countries with higher maternal death rates tend to have inadequate healthcare services for pregnant women and women who have recently given birth (1). This scenario is certainly aggravated when the healthcare system is overburdened, as it has been in the ongoing COVID-19 pandemic.

In Brazil, the maternal death rate has remained high in recent years. In 2019, 1,576 maternal deaths were recorded (2), which corresponds to a maternal death rate of 55.3/100,000 live births (3), demonstrating the fragility of our healthcare system when caring for this specific population.

Brazil has an influenza epidemiological surveillance information system [Sistema de Informação da Vigilância Epidemiológica da Gripe (SIVEP-Gripe)], a nationwide surveillance database created in 2009 and used to monitor severe acute respiratory infections (SARIs). As of 2020, the surveillance of COVID-19 has been incorporated into the SIVEP-Gripe database, which is updated weekly by the Ministry of Health through Open Data SUS [Sistema Único de Saúde] (4,5) (https://opendatasus.saude.gov.br/).

The Sivep-Influenza database provides information regarding confirmed cases, presenting a real-time analysis of this data is extremely important to government officials and all interested parties. Such data would increase the visibility of the status of pregnant women and women who have recently given birth in the setting of COVID-19 and provide guidelines for future public policies. With this in mind, on April 7, 2021, we created the Brazilian Obstetric Observatory (OOBr) COVID-19, a public dashboard that is updated weekly when new data are released by the Ministry of Health. Numerous exploratory data analyses are available on the OOBr website, with dynamic visualization and filtering, which can be customized by the user. The OOBr COVID-19 database can be accessed at https://observatorioobstetrico.shinyapps.io/covid_gesta_puerp_br (in Brazilian Portuguese) (6).

The OOBr COVID-19 is part of the Brazilian Obstetric Observatory, a project that analyzes nationwide public databases, such as live birth records (Live Birth Information System) and maternal and child mortality (Mortality Information System), with the objective of providing an interactive monitoring platform with scientifically based analyses, disseminating relevant information regarding maternal and child health.

Data were gathered from a period spanning from March 1, 2020 to May 5, 2021, at which point the database was downloaded. There were 1,184,365 confirmed cases of SARIs because of COVID-19. Of these, 11,247 (1%) cases occurred in pregnant or puerperal women. One significant fact is that, despite the pregnant and puerperal women residing in 1,954 different municipalities, hospitalizations were concentrated in only 1,046. These data indicate the possibility that pregnant women were willing to travel to obtain adequate care, which may be one of the factors influencing the prognoses of such cases.

During the period included for analysis, when taking into account only completed forms (hospital discharges or in-hospital deaths), there were 1,031 maternal deaths: 680 deaths were reported among pregnant women and 351 among postpartum women. COVID-19-induced SARIs in pregnant women peaked in the third trimester (4,505 cases), although mortality was the highest during the second trimester (214/1,873; 11.4%) and puerperal period (351/1,862; 18.9%). It is worth mentioning that 209/929 (22.5%) of the pregnant or puerperal women who died did not have access to an intensive care unit (ICU), and 307/917 (33.5%) did not have access to invasive ventilatory support.

When comparing Brazilian states, the inequality in access to healthcare was striking. The mortality rate in ICUs (Figure 1) ranged from 20.3% to 88.3%, the lack of access to an ICU (Figure 2) varied from 0 to 50%, and the lack of invasive ventilation ranged from 0% to 51.5% (Figure 3).

With the data gathered, an additional challenge presented itself. A comparison of data from 2020 and 2021 showed that the number of maternal deaths increased from 10.1 per week (456 deaths in 45 weeks in 2020) to 33.8 deaths per week (575 deaths in 17 weeks in 2021). This represents a 233.8% increase in the number of deaths per week in pregnant and postpartum women, a value much higher than that observed in the general population (97% increase). Therefore, a warning is in order. It is possible that severe acute respiratory syndrome coronavirus 2 variant P.1, which is increasingly prevalent in Brazil, may be associated with greater transmissibility, overloaded healthcare systems, and increased mortality, which may further increase maternal mortality rates.

## AUTHOR CONTRIBUTIONS

Francisco RPV and Rodrigues AS contributed to the study conception for the Brazilian Obstetric Observatory COVID-19 as well as to the composition of the manuscript. Lacerda L contributed to the construction and maintenance of the Brazilian Obstetric Observatory.

## Figures and Tables

**Figure 1:**
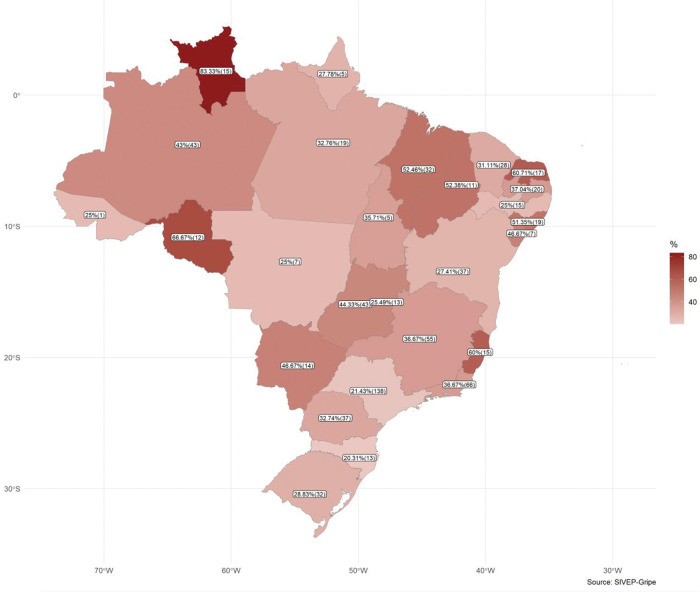
Deaths of pregnant and postpartum women with confirmed coronavirus disease (COVID-19) in intensive care units.

**Figure 2:**
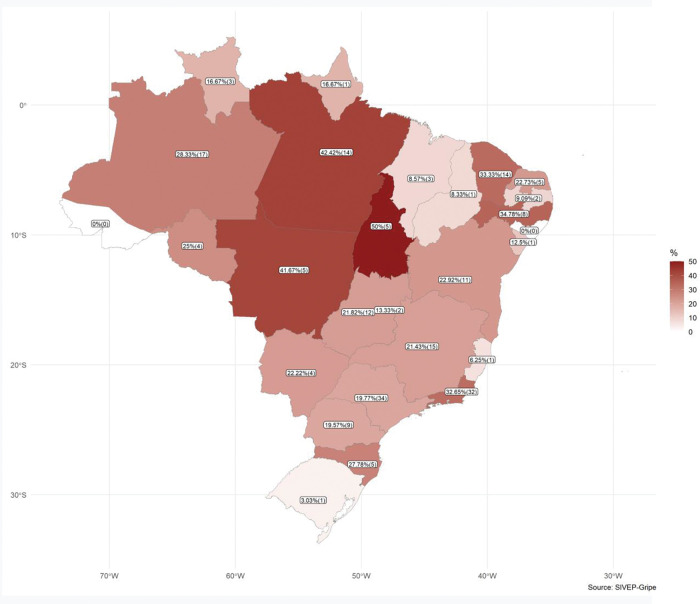
Non-admission of pregnant and postpartum women with confirmed coronavirus disease (COVID-19) to intensive care units.

**Figure 3:**
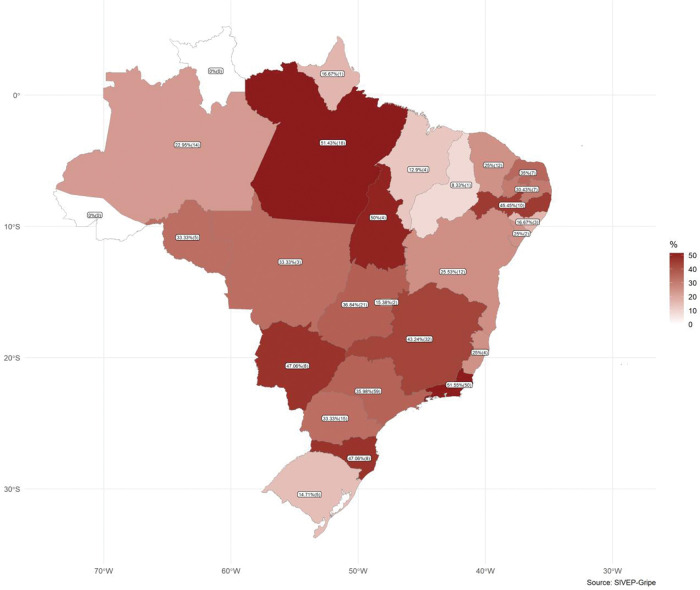
Non-orotracheal intubation in deaths of pregnant and postpartum women with coronavirus disease (COVID-19).
